# A Critical Analysis of Claims and Their Authenticity in Indian Drug Promotional Advertisements

**DOI:** 10.1155/2015/469147

**Published:** 2015-01-28

**Authors:** Gurpreet Kaur Randhawa, Navyug Raj Singh, Jaswant Rai, Gobindnoor Kaur, Resham Kashyap

**Affiliations:** Department of Pharmacology, Government Medical College, Amritsar 143001, India

## Abstract

*Introduction*. Drug promotional advertisements (DPAs) form a major marketing technique of pharmaceutical companies for promoting their products and disseminating ambiguous drug information which can affect prescribing pattern of physicians. Drug information includes product characteristics, various marketing claims with references in support to increase its credibility and authenticity. *Material and Methods*. An observational study was carried out on fifty printed drug advertisement brochures which were collected from different OPDs of Guru Nanak Dev Hospital attached to Government Medical College, Amritsar, India. These advertisements were analyzed and claims were categorized into true, false, exaggerated, vague, and controversial on criteria as reported by Rohraa et al. (2006). References of DPAs in support of the claims were critically analyzed for their retrievability from web and validity pertaining to claims. *Results*. Out of 209 claims from 50 advertisements, only 46% were found to be true, 21% false, 16% vague, 7% exaggerated, and 10% controversial in nature. Out of 160 references given in support of claims, 49 (30%) of references were irretrievable. Out of 111 (70%) retrievable references, 92 (83%) references were found valid. *Conclusion*. Drug information provided in the DPAs was biased, incomplete, unauthentic, and unreliable with references exhibiting questionable credibility.

## 1. Introduction

In 1930, a Drug Enquiry Committee was constituted by Sir Ram Nath Chopra in India which scrutinized the drug pamphlets making spurious claims much before WHO awakened to this threat in 1988 [1]. According to the “ethical criteria for medicinal drug promotion” by WHO, “drug promotion” refers to all informational and persuasive activities by manufacturers and distributors of the pharmaceutical industry, the effect of which is to induce a favorable prescription, supply, purchase, and/or use of medicinal drugs [2]. It includes activities of the medical representatives, drug advertisements and provision of gifts and free drug samples to prescribers, drug package inserts, direct-to-consumer advertisements, periodicals, telemarketing, holding of conferences, symposium, scientific meetings, sponsoring of medical education, and conduct of promotional trials [3]. Out of all, drug promotional advertisements (DPAs) form a major marketing technique of pharmaceutical companies for promoting their products and disseminating drug information for their own benefit. These advertisements disperse the information regarding product name and its pharmacological characteristics, price, marketing claims, and references cited in support of these claims. DPAs can be highly informative when it provides the authentic information in a nutshell as long as they have been critically appraised and reviewed [4].

Pharmaceutical companies spend around one third of all sales revenue on marketing their products which is twice that spent on research and development [5]. According to WHO, the global pharmaceuticals market is worth US$300 billion a year, a figure expected to rise to US$400 billion within the next three years [5]. In order to maintain the sales volume, there exists “an inherent conflict of interest between the legitimate business goals of manufacturers and the social, medical, and economic needs of providers and the public to select and use the drugs in the most rational way” [5].

Powerful influence of promotional advertisements on physicians prescribing behavior, dissemination of deceptive information, unsubstantiated claims, and lapses in the field of ethics is a matter of enormous concern worldwide for the past few decades. There is evidence that prescribers using the DPAs as the primary source of new information tend to prescribe less appropriately, hence compromising the patients' health in the process [6].

According to WHO, promotional claims need to be reliable, truthful, informative, balanced, up-to-date, and capable of substantiation of authentic information in good taste [7]. However, while the promotional methods have become very sophisticated and effective, the pharmaceutical companies do not adhere to the required ethical principles while promoting their products [3]. One of the vital features of drug advertisements is the references given in support of claims to increase the credibility and authenticity, but it has always been a grey area for manipulation by the pharmaceutical industry because of dearth of stringent guidelines for it in India. Section 4.2 of “Draft OPPI Code of Pharmaceutical Practices 2012” cautions against “absolute or all-embracing claims” and states that claims are made only with adequate qualification and substantiation [8]. On the part of prescriber, there is a need to “understand and respond” to the pharmaceutical promotional tactics and pressures in a much more responsible and diligent manner.

The objective of this study is to make the prescriber aware of the reliability and authenticity of the claims made in drug promotional literature, which is strategically placed in their hands by the medical representatives. Due care has been taken in scrutinizing the veracity of different claims in various drug advertisements. We also analysed the retrievability, validity, and credibility of references quoted in the DPAs.

## 2. Material and Methods

This was an observational study conducted by the department of pharmacology from January to March 2014. Seventy-five printed DPAs were collected from the prescribers in the Outpatient Departments of Medicine, Surgery, Orthopedics, and Psychiatry of Guru Nanak Dev Hospital attached to the Government Medical College, Amritsar, Punjab, India. These DPAs had been provided to the prescribers by medical representatives of various drug companies. A total of fifty DPAs were selected for analysis while those promoting medical devices (equipments, orthopedics, and prosthesis), Ayurvedic medicines, drug monographs, reminder advertisements, and identical advertisements were excluded from the present study. Prescribing information leaflets were also excluded as they were considered to be nonpromotional in nature [9–11].

These advertisements were analyzed critically and claims made therein were categorized based upon documented evidence cited in support and standard pharmacology textbooks like Goodman & Gilman's The Pharmacological basis of Therapeutics and Katzung's Basic and Clinical Pharmacology. Claims were categorized into true, false, ambiguous, exaggerated and controversial [9]. These five categories of claims were defined as follows.

1. True: a claim found to be completely justified according to the reference or evidence quoted in support.
2. False: a claim objectively incorrect and contradictory to evidence/cited reference or without any substantial evidence [12].
3. Ambiguous: a claim found to be vague in its description.
4. Exaggerated: a claim although not contradicting to evidence but out of proportion or overstated in comparison to the evidence or reference cited.
5. Controversial: a claim was defined as controversial when its content was still debatable and contentious in nature.

References quoted in DPAs were scrutinized for their retrievability from the web and their validity pertaining to claims. Each reference was traced using all available databases which included all indexed and nonindexed journals, PubMed, MEDLINE, and other web search engines. In case of any inaccessibility of full paper, their abstracts were retrieved. A reference was adjudged to be nonretrievable only when it was either not available in any database or could not be accessed because of missing or misprinting of one or more of the following requirements of standard bibliographic reference: author's name, title of article, journal's title/name, year of publication, issue and volume or supplement number and page numbers, book title, and publisher, if applicable. The references in DPAs were considered valid when the factual information in these references was comparable to the claims made and justified it. Partially valid references were also included under valid references (e.g., references explaining the ambiguous claims) whereas a reference was adjudged invalid if reference cited for the claim did not vindicate it.

## 3. Results

**Figure 1:**
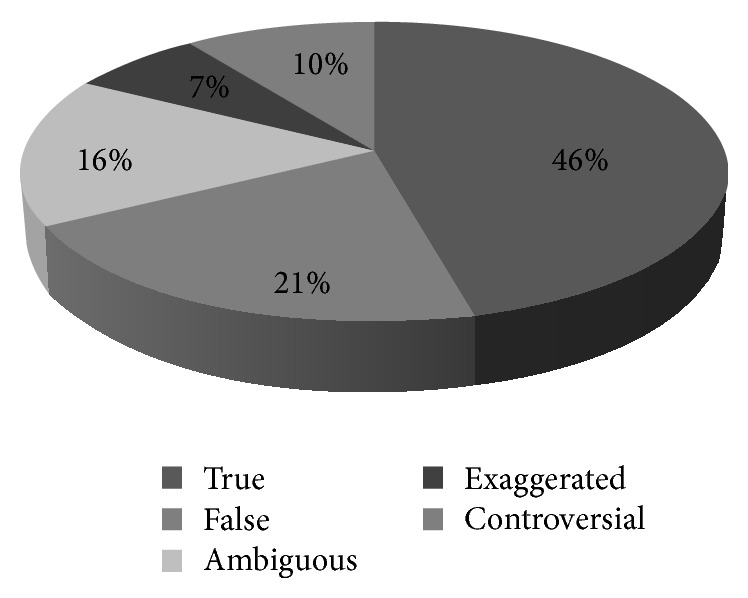
Categorization of claims in drug promotional advertisements.

**Figure 2:**
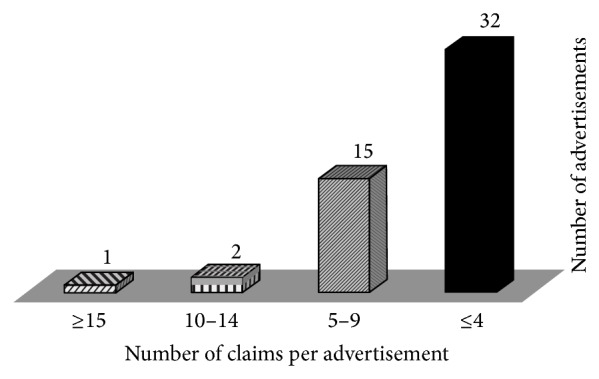
Graph depicting variation in number of claims per advertisement.

Fifty DPAs collected were published by thirty different pharmaceutical companies. 209 claims were found from 50 DPAs, which were supported by 160 references. Out of 209 claims, only 97 (46%) claims were found to be true while 54% claims were false (Table 1), ambiguous (Table 2), exaggerated (Table 3), and controversial (Table 4) in nature (Figure 1). It was also observed that the number of claims varied from one to seventeen per advertisement (Figure 2).

Claims in DPAs were also assessed in accordance with the pharmacological properties of the advertised drugs and their various clinical outcomes. 46% of the claims pertained to clinical efficacy with only 1% claiming safety and 24% for other pharmacological properties.

**Figure 3:**
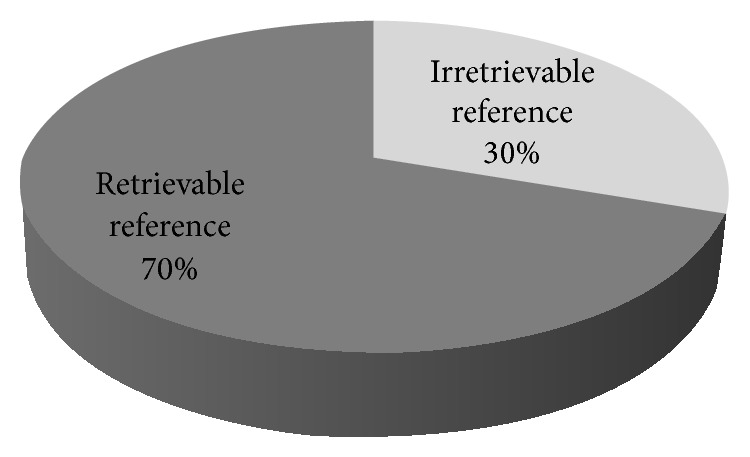
Classification of references as per retrievability pattern.

Cited references in support of the claims were also analyzed based on their retrievability pattern and validity arrays. Out of 50 advertisements, fifteen (30%) advertisements were without any reference. A total of 160 references were found from 35 advertisements. On the basis of retrievability pattern of cited references, it was observed that 70% of references were retrievable and out of them 17% were invalid (Figure 3).

Valid references among the 70% of retrievable ones included 83% of research articles which were good sources of authentic information like original research articles in indexed journals, though no meta-analysis was found to be referred (Tables 5 and 6).

## 4. Discussion 

The present study was an attempt to analyze DPAs from various drug companies in a tertiary care hospital of north India. On evaluation, it was observed that the DPAs were full of unsubstantiated claims with references being mentioned just to create an impression of being evidence based literature. Rather, only 46% of claims were found to be true and 54% of total claims being unjustifiable and similar findings were also observed by Villanueva and colleagues [13]. Claims outnumbered the references given in the promotional literature with 209 claims being supported by only 160 references which was less in comparison with other studies [10]. Apart from this, it was observed that on the one hand claims were supported by ten to seventeen references per brochure in 10% of the advertisements whereas claims in 30% of brochures lacked substantiation by a reference, which was quite similar to the results in other studies [10, 14]. Many of the ambiguous claims were having an intangible characteristic of using captivating phrases and statements which were found without any underpinning evidence but were capable of creating a compelling interest to prescribe. Such an influence is termed as “Red-Herring” effects which is defined as statements being used that have no link or association with clinical effectiveness of drug or statements with unique property of drug that may have no relevance to the therapeutic effect [15]. Out of 33 ambiguous claims, 20 were found proclaiming larger than life phrases like “best proven choice,” “most prescribed molecule worldwide,” and “remarkably safe,” hence exhibiting the Red-Herring effect, hence ascertaining the belief that the pharmaceutical companies are blatantly exploiting the biomedical literature to substantiate the ambiguous claims in support of their products [16].

It was observed that the claims in 70% of the cases laid emphasis on the efficacy and superiority while clinically relevant safety outcomes were negligibly (1%) highlighted. Similar findings mentioning the safety outcomes (37.2%) were also observed in another study [10]. Pharmaceutical companies have therefore deliberately highlighted the positive aspects of their products while hiding the negative aspects leading to the emergence of bias and thus translating into irrational prescribing behavior of the physician. Hence, irrational prescribing can lead to potential negative health consequences like treatment failures from the use of inappropriate drugs, unnecessary adverse effects, increase in antibiotic-resistant microorganisms, and an escalation in national health care costs [9].

Authentication of the claims by the biomedical research in the form of references seemed impressive initially with 111 retrievable references (70%). But the actual scenario was completely different with 209 claims found to be supported by only 92 (58%) valid references pertaining to claims from a total of 160 references quoted in DPAs. 49 references (30%) were of either dubious nature or were not retrievable. Hence, a large number of the references which were cited to increase the credibility of the DPAs were unjustified when they were critically analyzed. In some cases, the cited references led to articles published in non-English language journals and the translated abstract or full text articles of the same were unavailable. Three references were observed with the above-mentioned problem, while another three references were found to have a typographical error. On the quality assessment of evidence, majority of it pertained to original research articles (81%) with no meta-analysis or systematic review articles found in support. However, this finding was found to be different from another study by Cooper and Schriger [17]. Charan et al. (2011) found that among the drug advertisements published in the Indian medical journals, only 28% claims were supported by references and the most common references were of journal articles (75%) [18].

There were certain limitations of the present study like restricting to only one type of the promotional activity of the pharmaceutical companies (printed promotional literature) and also analyzing a small number (50) of drug advertisements. But the trend of strengths and weaknesses of DPAs can be assessed.

## 5. Conclusion

We conclude that the drug information provided in the DPAs can be biased, incomplete, unauthentic, and unreliable with references exhibiting questionable credibility. These might not help the physician to arrive at evidence based good prescribing decisions. Physicians need to be aware of the ambiguity in the information provided by DPAs and should be cautious not to rely solely on these. Awareness campaigns could be carried out for prescribers regarding deceptive information disseminated through DPAs.

Various strategies could be adopted to remain abreast of information while saving the time in the process. Making and regularly updating a list of personal drugs based upon WHO P-drug concept, asking for original and authentic sources of information regarding claims and treating the DPAs only as alerts to new developments, are some of the strategies which the physician can adopt. A few suggested sources of independent, authentic, and unbiased information [34] for the prescriber are like

1. International Society of Drug Bulletins, available at http://www.isdbweb.org/,
2. The Medical Letter, available at http://secure.medicalletter.org/,
3. Prescrire International, available at http://english.prescrire.org/en/,
4. Drug and Therapeutics Bulletin, available at http://www.dtb.org.uk/,
5. Prescriber's Letter, available at http://prescribersletter.therapeuticresearch.com/,
6. Worst Pills, Best Pills, available at http://worstpills.org/.

## Figures and Tables

**Table 1:** Examples of false claims made in drug advertisements.

1	Tiapride	Claim	61% improvement in cognitive impairment with tiapride as compared to 26.3% with quetiapine [19]
		Analysis	The claim was based on a referenced study which dealt with tiapride versus haloperidol and tiapride versus placebo in elderly patients with cognitive impairment and not quetiapine

2	Combination of thiocolchicoside, aceclofenac, and paracetamol	Claim	Thiocolchicoside is safe and nonsedating muscle relaxant
		Analysis	(i) Primary adverse effects with thiocolchicoside include somnolence, vasovagal attack, and hepatic toxicity [20] (ii) There is no reference in support of this claim

3	Rabeprazole plus diclofenac	Claim	Rabeprazole is ideal for prophylactic use in NSAIDS in high risk patients
		Analysis	(i) Lansoprazole has been recommended in high risk patients prone to ulcers and not rabeprazole [21, 22] (ii) Irretrievable reference

4	Diclofenac	Claim	Lesser incidence of GI toxicity than nonselective NSAIDS such as indomethacin
		Analysis	GI symptoms are most common adverse effects observed with diclofenac with about 2% people withdrawing from treatment due to these side effects [23]

**Table 2:** Examples of ambiguous claims in drug advertisements.

1	Nitroglycerin	Claim	An optimal clinical response for improved quality of life (Qol)
		Analysis	(i) Claim did not specify the type/kind of clinical response and how it improved the quality of life (ii) No reference cited in support

2	Combination of carbonyl iron/vitamin B12/vitamin C/vitamin E/folic acid/sodium selenite/copper sulphate and zinc sulphate	Claim	Slow rate of solubilization resulting in gentle absorption
		Analysis	(i) No pharmaceutical data provided (ii) No reference mentioned in support

3	Chlorothiazide plus Telmisartan	Claim	C the difference with better partner
		Analysis	(i) Incomprehensible phrase without any reference quoted in support

4	Ivabradine	Claim	Decrease the need for hospitalizations, emergency services, and sick leaves versus usual care
		Analysis	(i) Unable to explain how the referred drug decreased the morbidity (ii) Irretrievable reference in support

5	Esomeprazole	Claim	The most prescribed proton pump inhibitor (PPI) worldwide
		Analysis	(i) Vague claim without any reference

**Table 3:** Examples of exaggerated claims in drug advertisements.

1	Perindopril plus amlodipine	Claim	Perindopril/amlodipine with stronger and superior BP reduction as compared to telmisartan/amlodipine and olmesartan/amlodipine
		Analysis	The referenced study dealt with the efficacy of perindopril/amlodipine as antihypertensive agents only, with no comparison to other drug combinations [24, 25]

2	Moxifloxacin	Claim	Used in Multidrug Resistant TB
		Analysis	Approved by FDA for nontubercular infections but it is under phase 3 trials for use in tuberculosis [26]

3	Vitamin D3	Claim	Increases Bone Mineral Density (BMD) by 25% within 2 years
		Analysis	Referenced study affirmed increase in BMD on vitamin D3 therapy but not by 25% in 2 years [27]

4	Duloxetine	Claim	Offers advantage in terms of efficacy over SSRIs
		Analysis	(i) No significant advantage has been seen in comparison to SSRIs [28, 29] (ii) SSRIs are the most commonly prescribed 1st line agents in treatment of anxiety and major depressive disorder due to their better safety and efficacy profile [28, 29] (iii) No reference was quoted in support

**Table 4:** Examples of controversial claims in drug advertisements.

1	Methylcobalamin	Claim	Role in neuropathic pain as powerful rejuvenator, enhancing nucleic acid proteins and myelin sheath [30]
		Analysis	The referenced study was an experimental one in rats and not in humans

2	Cefuroxime plus clavulanic acid	Claim	Superior beta lactamase inhibition compared to tazobactam and sulbactam
		Analysis	(i) Sulbactam, tazobactam, and clavulanic acid are found to be equally efficacious against beta-lactams [30] (ii) Efficacy regarding mentioned combination for beta lactamase inhibition not found in medical literature (iii) No reference was quoted for the claim

3	Nebivolol	Claim	Decreases triglycerides and increases HDL
		Analysis	(i) Exact effect of nebivolol or its mechanism on lipid profile is still not known [31] (ii) No reference quoted for the claim

4	Deflazacort	Claim	Minimal effect on HPA axis suppression compared to prednisolone [17]
		Analysis	The referenced study could not explain how and why deflazacort, a corticosteroid like prednisolone, has minimal suppression on HPA axis

5	Alendronate	Claim	No significant excess risk of fracture on prolonged use
		Analysis	(i) Risk of subtrochanteric and diaphyseal stress fractures of femur in patients taking alendronate for >5 years was increased from 13 in untreated women to 31 per 10,000 patient-years in treated women [32]. (ii) Benefit of alendronate in preventing osteoporotic fractures by 34% was seen in another study [33]. (iii) No reference cited in support

**Table 5:** Classification of retrievable references.

Types of reference	Valid (*n*)	Invalid (*n*)
^*^Research article	75	15
Review article	5	—
Meta-analysis	—	—
Preclinical studies		
Animal studies	—	—
In vitro studies	—	1
Book	—	1
Website	2	—
Data on file	—	—
^**^Other journal articles	2	1
^***^Other references	8	1

Total retrievable references = 111	92	19

^*^Research article includes randomized controlled trials, randomized placebo controlled trials, nonrandomized trials, clinical trials without details of design, observational studies without details of design, retrospective studies, case-control studies, and postmarketing surveillance studies.

^**^Other journal articles: case report, correspondence article, editorial, and letter to the editor.

^***^Other references: conference proceedings, report, departmental study, therapeutic guidelines, newspaper article, health magazine article, unpublished trial or surveillance data, online medicine prescription information, physicians' desk reference, pharma-aid, and reference with vague description.

*n*: Number of references.

**Table 6:** Evaluation of references on irretrievability pattern.

References not retrievable	43
Journal citation with typographical error	3
Journal in other languages	3

Total irretrievable references	49 (30%)
